# Voice-Enabled Virtual Patients for Interactive Training in Standardized Clinical Assessment: Mixed Methods Pilot Study

**DOI:** 10.2196/89066

**Published:** 2026-10-02

**Authors:** Veronica Bossio Botero, Vijay Yadav, Jacob Ouyang, Anzar Abbas, Michelle Worthington

**Affiliations:** 1 Brooklyn Health, 636 Broadway, Suite 301, New York, NY, 11201, United States, 1 917-284-8568; 2 School of Psychology, University of New South Wales, Sydney, NA, Australia; 3 Department of Psychiatry, Yale School of Medicine, New Haven, CT, United States

**Keywords:** virtual patient, large language models, clinical simulation, medical education, psychiatry, depression, artificial intelligence, AI

## Abstract

**Background:**

Training mental health clinicians to conduct standardized clinical assessments is challenging due to a lack of scalable, realistic practice opportunities. Traditional methods often fail to prepare trainees for the variability and complexity of real-world patient interactions, potentially impacting data quality in clinical trials. This paper introduces a novel approach to address this training gap using large language model (LLM)–based interview simulations.

**Objective:**

This study aimed to develop and validate a voice-enabled virtual patient simulation system as a proof of concept. We described the development of the system and evaluated whether it could generate virtual patients that (1) accurately adhered to predefined clinical profiles, (2) maintained a coherent and consistent narrative, and (3) produced dialogue that is perceived as realistic.

**Methods:**

We implemented a system that used an LLM to simulate patients with specified symptom profiles, demographic backgrounds, and distinct communication styles. The system’s performance was analyzed through a mixed methods evaluation, which included a formal assessment by 5 experienced clinical raters who conducted simulated structured Montgomery-Åsberg Depression Rating Scale (MADRS) interviews with 4 virtual patient personae, scored them on the scale, and provided qualitative feedback on the system’s clinical plausibility, narrative cohesion, and dialogue realism.

**Results:**

Across 20 interviews, the virtual patients demonstrated reasonable adherence to their configured clinical profiles, with human rater scores falling near their predefined score configurations. The mean item difference between MADRS rater scores and configured scores was 0.52 (SD 0.75); interrater reliability for the total score was 0.90 (95% CI 0.68‐0.99). Expert raters consistently gave average ratings of “agree” to “strongly agree” when asked to evaluate the qualitative realism and cohesion of the virtual patients.

**Conclusions:**

LLM-powered virtual patient simulations offered a promising, scalable tool for training clinicians in standardized clinical assessment. This pilot study provides initial evidence for the system’s ability to produce clinically relevant practice scenarios with reasonable fidelity.

## Introduction

The clinical interview is a fundamental tool in psychiatric assessment and serves as a primary endpoint in clinical trials for central nervous system (CNS) disorders. Because most psychiatric conditions lack objective biomarkers, the field relies on subjective clinical evaluations. The validity of clinical trials, and consequently, the development of new therapeutics, thus fundamentally depends on the quality of these assessments. Despite its critical role, the development of consistent interviewing skills remains a significant challenge, as conventional training methods frequently fail to provide scalable and realistic practice environments for this complex task.

High interrater reliability in clinical assessments is essential for minimizing data noise and identifying the true effects of treatment. However, extensive evidence indicates that conventional clinical rater training approaches, such as peer role-play, are insufficient for achieving this standard, showing little to no improvement in the validity of trainee assessments [1-3]. Even when initial training is successful, rater scores often shift over the course of a trial, a phenomenon known as “rater drift,” which can further compromise data quality [2,4,5]. At the core of this issue is a fundamental methodological gap: the lack of scalable, on demand, and standardized “patient stimuli” to calibrate and maintain rater performance over time.

Current methods for clinical interview training include watching prerecorded videos or conducting role-play with peers, both of which fail to replicate the realism and interactive nature of real clinical scenarios. The certification process for clinical trial raters introduces a further systemic obstacle. Sponsors typically require raters to meet strict criteria, often including a minimum number of completed scale administrations. Because uncertified raters cannot access opportunities to conduct assessments, they cannot meet the experience thresholds required for certification. This circular barrier limits the pool of trained raters and perpetuates poor measurement practices.

The use of simulation to bridge the gap between training and clinical practice has been an important tool in medical education for decades, with applications in many specialties [6-8]. Standardized patients—individuals trained to consistently portray specific clinical scenarios—provide a more realistic alternative to peer role-play for teaching communication and diagnostic skills. However, they have significant limitations, as they are expensive and difficult to scale, which limits the repeated practice opportunities that are essential for skill development [9]. Moreover, human variability in standardized patient performance can compromise the very standardization they aim to achieve [10,11].

To address these limitations, the field has developed successive generations of virtual patients—computer systems designed to simulate clinical scenarios. While virtual patients evolved from simple branching narratives to being capable of processing free-text input, their reliance on matching user inputs to a fixed database of prescripted responses was a fundamental limitation [12-14]. Consequently, the trade-off between the scalability of automated systems and the realism of standardized patients has persisted.

Large language models (LLMs) offer a solution to this long-standing challenge by shifting the mechanism of simulation from information retrieval to the generation of novel content. By synthesizing context-aware language in real time, these models are able to simulate standardized clinical scenarios while maintaining flexibility and realism. Emerging empirical evidence supports this utility: randomized trials show that LLM-based simulations achieve high dialogue authenticity ratings and reduce trainee anxiety compared with live observation, providing an accessible environment for skill development [15-19]. As Zeng et al [20] noted in a 2025 scoping review, while LLM-based virtual patients are still in the early stages of development, they hold significant potential for improving medical training.

Here, we describe the development and initial evaluation of a voice-enabled virtual patient system designed specifically for CNS clinical trials. The system uses a full speech-to-speech pipeline to simulate highly interactive and realistic clinical assessments. As an initial evaluation of the system, we tested the alignment of expert-rated symptom severity scores on the Montgomery-Åsberg Depression Rating Scale (MADRS) [21] with predefined scores for virtual patient personae. The primary objectives of this pilot study were to determine whether the system could generate virtual patients that (1) adhere to preconfigured clinical profiles with sufficient accuracy for expert raters to score them reliably, (2) maintain a coherent and consistent narrative throughout an interview, and (3) produce dialogue that clinical experts perceive as realistic.

## Methods

### System Design

Our system provides a simulated clinical encounter where a user can practice conducting an interview-based clinical assessment with a virtual patient using a speech-to-speech pipeline. The system’s architecture (Figure 1) is composed of 2 primary components: a patient profile engine for constructing clinically plausible and narratively coherent personae dynamically, and an interactive voice pipeline that animates these personas through real-time, spoken dialogue.

**Figure 1.**
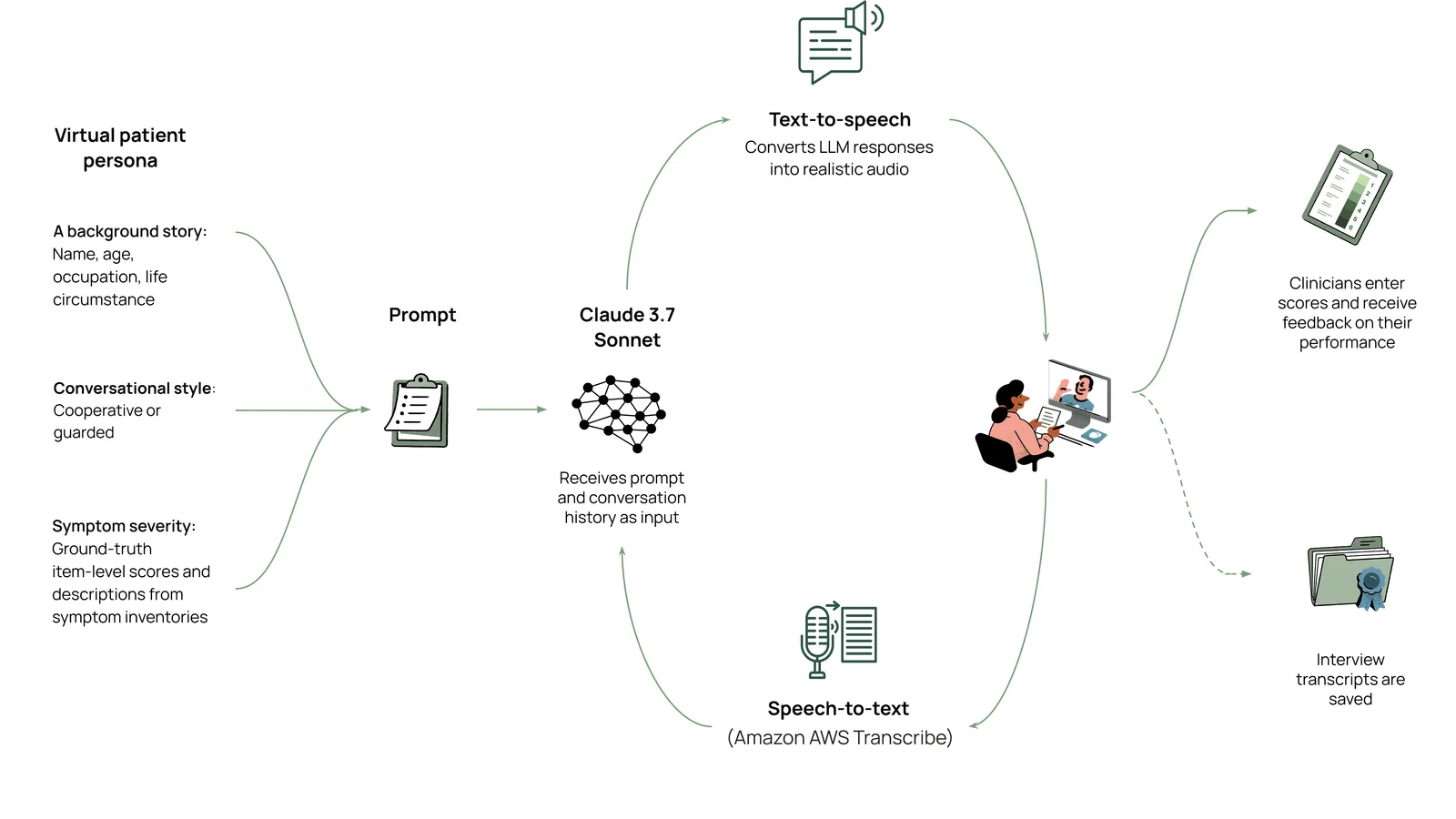
System architecture and interaction workflow. The clinician’s speech is transcribed by a speech-to-text module. The resulting text is processed by a large language model (LLM), which generates a response grounded in a detailed system prompt (defined by the patient’s symptoms, background, and style). A text-to-speech model synthesizes the LLM’s response into audio, completing the real-time, turn-based dialogue loop.

### Patient Profile Generation

The core of each profile is a randomly generated set of scores for the symptoms defined by the MADRS, which collectively define the clinical presentation. Importantly, this random generation was constrained by logical consistency checks adapted from the International Society for CNS Clinical Trials and Methodology (ISCTM) working group algorithms to ensure that the co-occurrence of symptoms is clinically plausible [22]. For example, a high score for suicidal ideation must be accompanied by a high score for depressed mood, and items reflecting overlapping symptom domains (eg, reduced sleep and lassitude) are constrained to mutually consistent ranges. Profiles failing any consistency checks are regenerated prior to presentation.

In addition to the clinical profile, each virtual patient is assigned a specific communication style that modulates how their symptoms are communicated. The styles are cooperative, a baseline style where responses are designed to provide clear and scorable information; and guarded, which reflects the communication barriers often present in depression, where a patient may be reluctant to disclose details. Finally, each profile is contextualized within a unique demographic and situational narrative, including a name, age, and personal history. Demographic and situational narratives were generated to provide contextual coherence for each persona rather than as systematically controlled experimental variables. Race, ethnicity, and socioeconomic status were not explicitly specified or balanced across personae in this pilot study.

The target item scores and their corresponding behavioral descriptions are combined with the demographic information and the communication style into a detailed system prompt. This prompt structure serves as the complete specification for the virtual patient’s persona and is provided to the core LLM (Anthropic’s Claude 3.7 Sonnet [23]) to guide its dialogue generation throughout the interview, ensuring each interaction is grounded in a consistent and clinically valid profile. During development, observed failure cases (eg, dialogue drifting toward a symptom profile inconsistent with the configuration) informed iterative refinement of the prompt structure prior to the user study, although runtime detection of failure cases was not included as part of this study.

### Interactive Voice Pipeline

The interactive dialogue is driven by a real-time speech-to-speech pipeline. The loop begins with the clinician’s spoken input, which is captured and transcribed using Amazon Transcribe’s streaming speech-to-text (STT) service [24]. The resulting text is then passed to the language model, which generates a response based on the patient profile and the evolving conversation history.

This response is then converted back into speech using a text-to-speech (TTS) model. The system architecture supports several high-fidelity TTS providers—including Amazon Polly, ElevenLabs, and Cartesia—allowing for flexible control over vocal characteristics and degrees of realism. For the present study, ElevenLabs voice models were used exclusively. The interview continues until the clinician determines they have sufficient information for a clinical assessment, at which point their scores, qualitative feedback, and the interview transcript are recorded.

### User Study

To evaluate the system’s performance and perceived quality, we conducted a user study with trained clinicians who had extensive experience administering the MADRS. The study was designed to assess the virtual patients’ adherence to their clinical profiles by testing the alignment of the rater-assigned MADRS scores with the predefined MADRS scores and to gather expert feedback on the realism and coherence of the simulated dialogue.

### Participants

A group of expert raters (n=5) was recruited through our professional network. Participants were required to have a master’s degree or higher in psychology, psychiatry, social work, or a related field and have prior experience administering the MADRS.

### Materials

We created a fixed set of 4 virtual patient “personae” for the study [25]. The personae were balanced for gender (2 men and 2 women) and communication style (2 cooperative and 2 guarded). The underlying preconfigured profiles were designed to span a wide range of symptom severities as defined by the MADRS, with total scores ranging from 14 (mild) to 39 (severe). All expert raters interacted with the same 4 virtual patients to ensure consistency and allow for interrater reliability analysis. The order of personae was randomized across raters to account for practice effects and fatigue effects.

The core dialogue engine was driven by Anthropic’s Claude 3.7 Sonnet LLM [23]. STT transcription was handled by Amazon Transcribe [24], and speech synthesis (TTS) was generated using ElevenLabs voice models [26].

### Procedure

The study was conducted remotely via video call, as opposed to a voice call, to facilitate study procedures and leverage the high quality of audio transmission across teleconferencing technology as compared with a phone call. After providing informed consent, each participant received a brief onboarding to demonstrate the system’s voice-based interface and to review the study tasks. Participants were instructed to administer the MADRS for each virtual patient, following the Structured Interview Guide for the MADRS (SIGMA) methodology [27], which typically takes 30 to 45 minutes to administer.

For each of the four personae, the procedure was as follows. First, the expert rater conducted the full voice-based interview with the virtual patient using the speech-to-speech system. Second, immediately following the interview, the rater was directed to a postinterview survey via a link through which deidentified responses were collected. Third, in the survey, the rater first provided their clinical scores for the virtual patient on each of the 10 MADRS items. Fourth, after submitting their scores, the rater was presented with a comparison chart showing their ratings alongside the virtual patient’s preprogrammed profile to inform the rater’s assessment of adherence to the clinical profile. They were then asked to complete the qualitative feedback portion of the survey.

### Measures

#### Clinical Scoring

Raters scored the virtual patient on each of the 10 items of the MADRS based on the evidence gathered during the interview.

#### Qualitative Ratings

Raters indicated their agreement on a 7-point Likert scale (1=strongly disagree to 7=strongly agree) with 3 statements. The first statement assessed profile consistency: “The behavior of the virtual patient during the interview was consistent with the symptom profile it was configured to exhibit.” As this question was presented after raters saw the preconfigured scores, it was not designed to re-evaluate scoring alignment (which was captured by the quantitative “clinical scoring”), but rather to serve as a meta-assessment of portrayal quality. Its purpose was to determine whether any observed discrepancies between the expert’s initial score and the simulation configuration were still perceived as falling within a clinically plausible range. Because raters viewed the comparison between their scores and the preconfigured scores prior to answering this question, the resulting rating is susceptible to anchoring and confirmation bias and should be interpreted as a complementary perceptual measure rather than an independent validation of portrayal fidelity.

### Ethical Considerations

This study was reviewed by the Biomedical Research Alliance of New York, protocol 25-196-2265, and was determined to be exempt from review. Given this exempt status, documentation of informed consent was waived; however, an informed consent script was read to each participant prior to participating. No identifying data were collected, and all study data were anonymized using a unique study ID for each participant. Participants did not receive any compensation.

## Results

### Alignment of Predefined and Rater-Scored MADRS Scores

Across all MADRS items, which are each rated on a 0 to 6 scale, the mean difference between rater and preconfigured scores was 0.52 (SD 0.75). The distributions of these item-level score differences were clustered around 0 but exhibited a consistent rightward skew (Figure 2), indicating that raters tended to assign slightly higher scores than the target scores. This trend was stable across all 4 personae, with mean item score differences of 0.60, 0.60, 0.66, and 0.46, respectively (mean SD across the 4 personae was 0.07; Figure 3).

**Figure 2.**
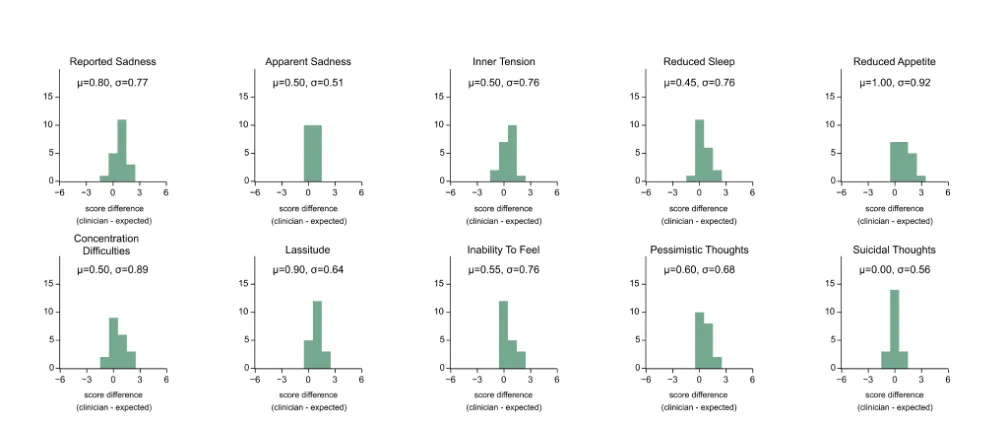
Average score differences (μ) between clinician score and preconfigured score for each Montgomery-Åsberg Depression Rating Scale item, shown for the 4 participant personae. Error bars represent the SEM (σ) across raters.

**Figure 3.**
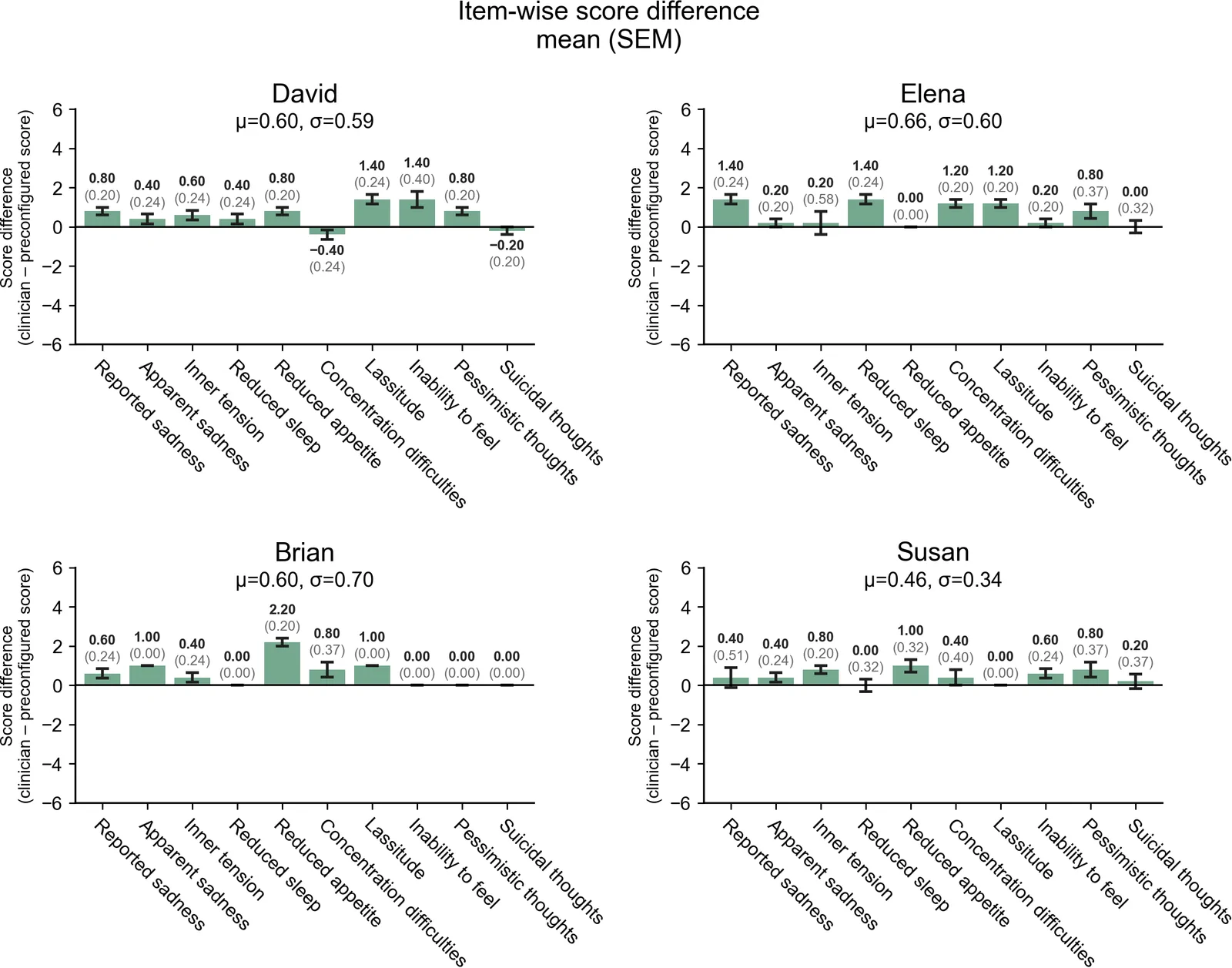
Average score differences between clinician score and preconfigured score for each Montgomery-Åsberg Depression Rating Scale item, shown for the 4 participant personae. Error bars represent the SEM across raters.

The cumulative effect of these small but systemic overestimations at the item level resulted in a consistent difference in the total MADRS score (the overall sum of the item-level scores). While the mean total MADRS scores assigned by raters tracked the intended severity across all personae (Figure 4), clinician scores averaged moderately higher than the configured ground truth values (mean absolute error 5.80, SD 0.73).

**Figure 4.**
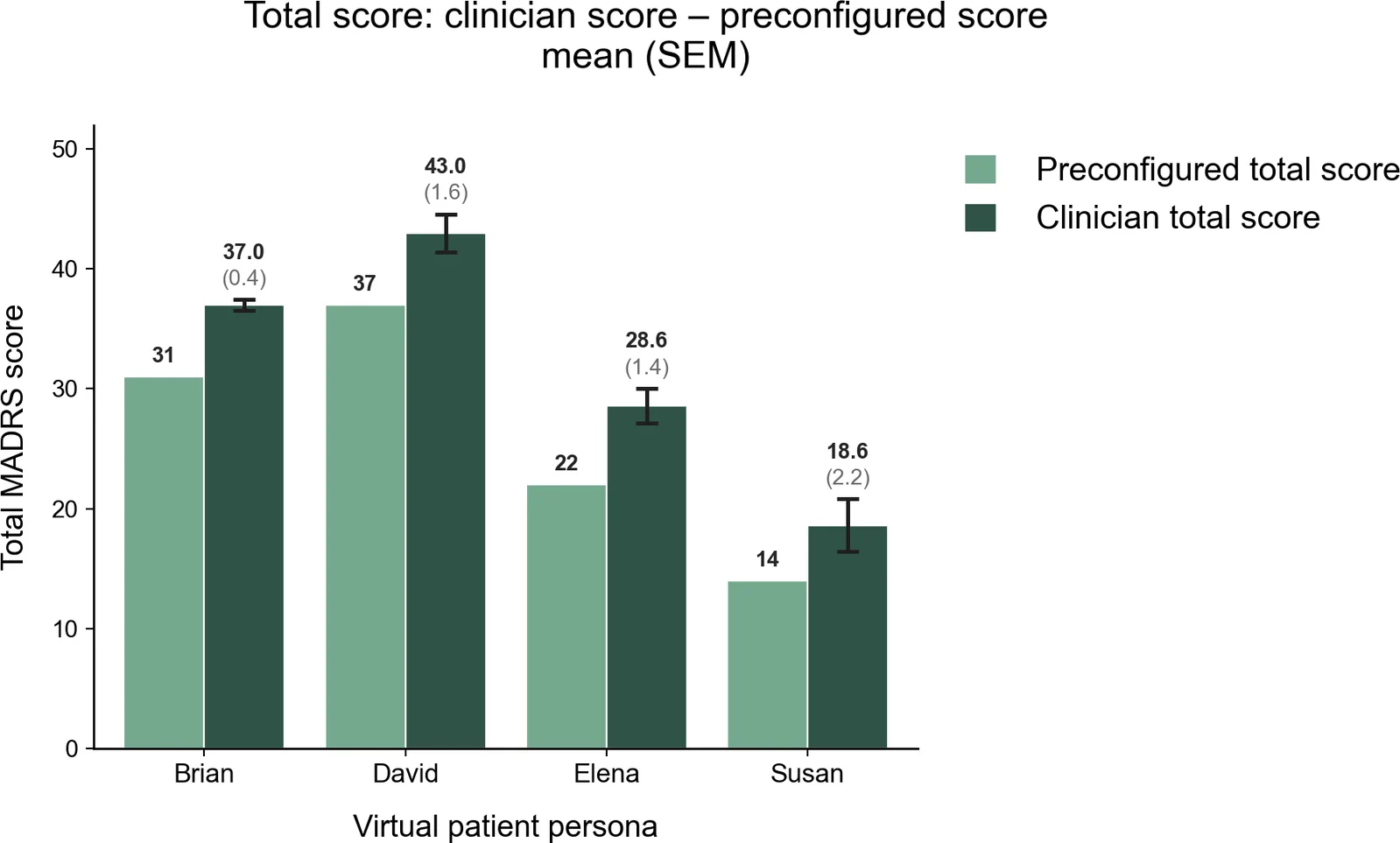
Total Montgomery-Åsberg Depression Rating Scale (MADRS) score from the virtual patient configuration (light green) versus total MADRS score assigned by the clinicians (dark green) for each of the 4 patient personae.

### Interrater Reliability Across Expert Raters

The virtual patients elicited consistent ratings across the raters in the study. Interrater reliability for the total MADRS score was high, with an intraclass correlation coefficient (ICC) of 0.90 (95% CI 0.68-0.99) based on a 2-way random-effects model for a single rater’s absolute agreement (ICC(2,1)). Reliability for individual items was variable. Agreement was excellent for “lassitude” (ICC 0.98, 95% CI 0.91-1.00), “reduced appetite” (ICC 0.96, 95% CI 0.86-1.00), “reduced sleep” (ICC 0.95, 95% CI 0.82-1.00), and “apparent sadness” (ICC 0.93, 95% CI 0.75-0.99); good for “suicidal thoughts” (ICC 0.88, 95% CI 0.62-0.99), “pessimistic thoughts” (ICC 0.85, 95% CI 0.53-0.99), “reported sadness” (ICC 0.77, 95% CI 0.41-0.98), and “inability to feel” (ICC 0.75, 95% CI 0.38-0.98); and moderate for “concentration difficulties” (ICC 0.69, 95% CI 0.29-0.97) and “inner tension” (ICC 0.63, 95% CI 0.20-0.96). CIs for individual items were wide, reflecting the small number of virtual patients rated, and interval width rather than point estimate accounts for much of the apparent spread across items.

### Assessing Profile Consistency, Coherence, and Realism

Qualitative ratings for the 20 interviews indicated broadly favorable perceptions of the system’s performance. When asked whether the patient’s behavior was consistent with its configured profile, the ratings for all interviews were positive, including “strongly agree” (n=5), “agree” (n=14), and “somewhat agree” (n=1; Figure 5B). In 19 out of the 20 interviews, the character cohesion was judged favorably, with ratings to the statement “The virtual patient maintained a coherent character throughout the interview, without factual or behavioral contradictions” including “strongly agree” (n=11), “agree” (n=4), and “somewhat agree” (n=4; Figure 5A). One rating was “somewhat disagree” (n=1), with the rater commenting that the virtual patient was factually consistent but at times behaviorally inconsistent. Ratings for linguistic naturalness were also high, with 19 of 20 responses being positive: “strongly agree” (n=9), “agree” (n=9), and “somewhat agree” (n=1; Figure 5C). One interview received a rating of “somewhat disagree” (n=1), for which the rater commented that the virtual patient was unrealistically verbose compared with a severely depressed person.

**Figure 5.**
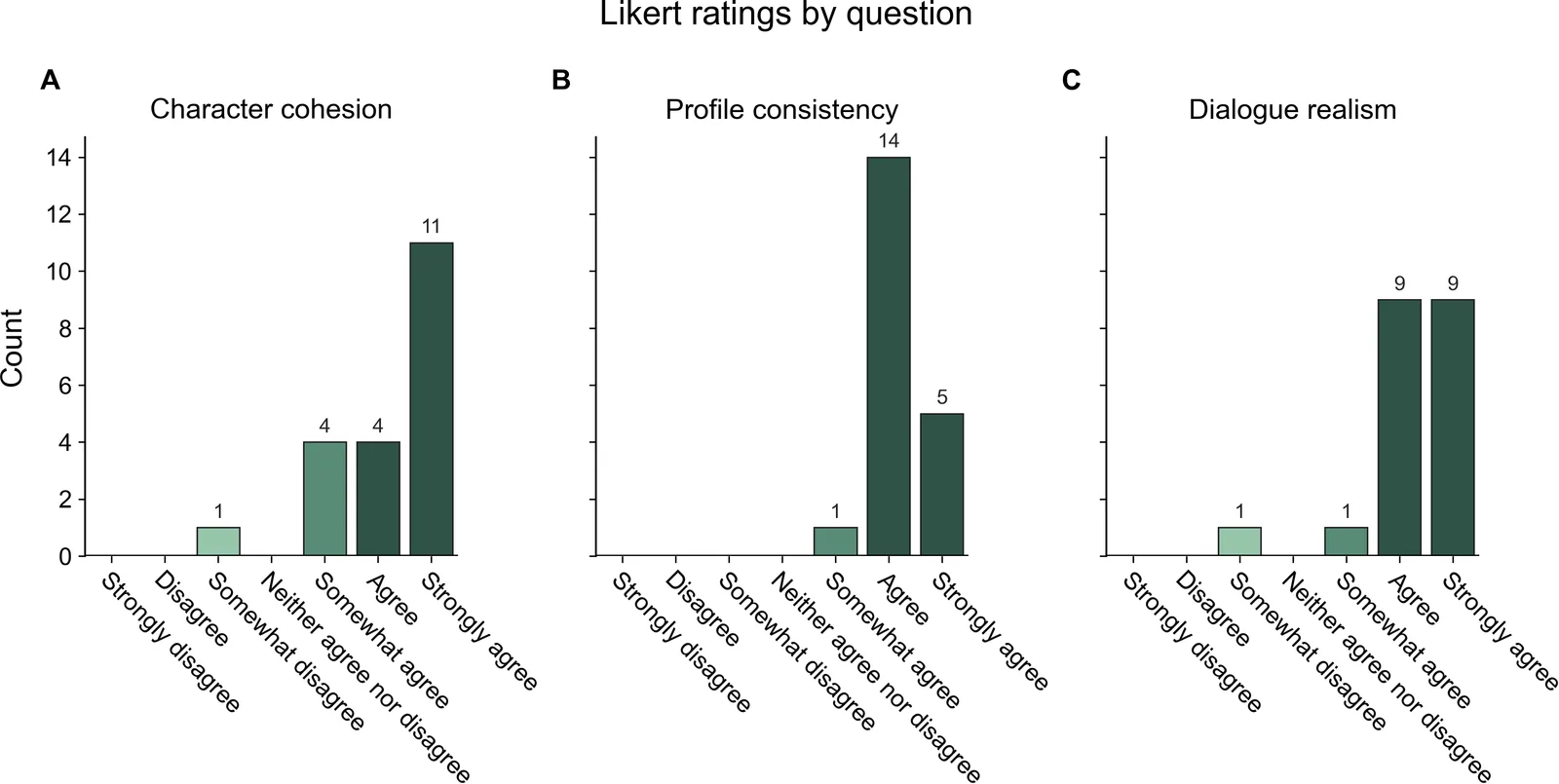
Likert-scale responses to the 3 qualitative questions across expert raters and interviews. A. Expert rater responses to prompt: "The virtual patient maintained a coherent character throughout the interview, without factual or behavioral contradictions." B. Expert rater responses to prompt: "The behavior of the virtual patient during the interview was consistent with the symptom profile it was configured to exhibit." C. Expert rater responses to prompt: "The dialogue produced by the virtual patient was realistic and natural in terms of language structure and vocabulary."

## Discussion

### Primary Findings

The results of this pilot study suggest that a voice-enabled virtual patient system based on an LLM can produce cohesive and clinically plausible simulations of interview-based clinical assessments. The primary findings show that experienced clinicians scored a set of virtual patients with reasonable fidelity to their predefined symptom profiles and that interrater reliability point estimates were promising. These results provide initial evidence that LLM-based simulations can serve as a standardized and scalable tool to address critical gaps in clinical interview training, particularly for CNS clinical trials.

A notable finding in our study was the systematic tendency for expert raters to score the virtual patients’ symptoms as moderately more severe than their predefined parameters, resulting in a mean absolute error of 5.80 on the total MADRS score. A difference of this magnitude is clinically meaningful and could shift a patient’s severity classification, so its source warrants careful consideration. One possibility is that the LLM, in its current configuration, generates richer or more evocative symptom descriptions than required to meet a given scoring threshold, leading raters to “round up.” Another plausible explanation is that the model is miscalibrated at the generation step: it may fail to translate configured severity scores into dialogue that distinguishes adjacent severity levels with sufficient precision, producing presentations that systematically overrepresent severity relative to the target. Our current data cannot distinguish between these mechanisms, and the true source is likely an interaction between model output characteristics and rater judgment. As shown in Figure 3, the upward bias was not uniform across items, suggesting that certain symptom domains may be more susceptible to either overgeneration by the model or systematic rater leniency. This presents a clear avenue for model refinement, possibly through modifications to the system’s prompting or by fine-tuning the model on a corpus of expert-scored interview transcripts to more precisely capture the subtle nuances of symptom expression at each severity level [28].

Our quantitative analysis yielded a high point estimate for the interrater reliability for the total MADRS score (ICC=0.90), which is a promising early signal, rather than a robust estimate, of the system’s ability to portray symptoms consistently. With only 5 raters and 4 personae, the variance components used to compute the ICC are based on a small number of observations, the CI is wide (0.68‐0.99), and the estimate is statistically unstable. This estimate demonstrates trend alignment with an ICC of 0.93 reported in the original validation of the structured interview guide (SIGMA) with human patients [27]; however, a more reliable and stable ICC estimate would require data from interviews with closer to 30 different patients. Item-level ICC point estimates are similarly preliminary and should be interpreted with the same caution. The variability in item-level reliability also mirrors findings in the SIGMA study, which characterized its item-level reliability as “good to excellent.” This suggests that such variability may reflect not just system-specific behavior but also the inherent ambiguity in rating certain symptoms. Further evaluations with a larger sample of raters and patient personae will be needed to assess this question.

The broadly positive qualitative feedback is an encouraging sign of the system’s fidelity, although these findings should also be considered preliminary, as the total number of interviews (n=20) is moderate. Raters agreed uniformly that the virtual patients’ behavior was consistent with their configured profiles, suggesting that even though experts’ blinded quantitative scores differed from the ground truth, they still perceived the behavior as a reasonable representation of the intended clinical profile. As noted in the Methods section, raters answered the profile-consistency item only after viewing the comparison between their own scores and preconfigured scores; these ratings are therefore susceptible to anchoring and confirmation bias and constitute a complementary perceptual measure rather than an independent validation of portrayal fidelity.

This observation is consistent with the strong ratings for character cohesion (19 of 20 positive) and dialogue realism (19 of 20 positive). The isolated “somewhat disagree” ratings are valuable, as they point to specific areas for refinement. For instance, one rater’s observation that despite factual consistency, the virtual patient appeared, at times, behaviorally inconsistent, highlights the challenge of maintaining nuanced, implicit manifestations of symptoms throughout the entire conversation. Another comment suggested that instances of unrealistic verbosity occurred specifically in the context of severe symptoms. This feedback directly suggests a need to calibrate the model’s output to better match this typical clinical presentation. However, these individual observations did not appear to detract from the overall expert agreement. Taken together, these results provide initial evidence that the tool is capable of producing interactions that are clinically consistent, factually and behaviorally cohesive, and linguistically natural.

These findings address long-standing limitations of current training paradigms. The system presented here offers a compelling alternative that combines the realism of standardized patients with the scalability and standardization afforded by automated solutions. By providing on demand access to a wide range of patient personae, the virtual patient simulations could offer trainees the repeated practice opportunities needed to build expertise. Furthermore, the system’s ability to generate consistent patient stimuli could be leveraged to calibrate raters and mitigate “rater drift” throughout the course of a clinical trial, contributing to enhanced data quality.

### Limitations and Future Directions

Despite the encouraging preliminary findings, this study has several limitations that highlight important directions for future research. The most critical limitation is the study’s sample size (4 virtual patient personae and 5 expert raters), which is appropriate for a pilot study but limits the broader generalizability of our findings. Notably, this small sample size precludes more in-depth subanalyses of demographic features, including gender, verbosity, and other characteristics not systematically controlled for in this study, such as race, ethnicity, education, and socioeconomic status. A larger-scale evaluation with more diverse patient characters and a larger set of raters is needed to validate the initial results from this pilot study.

The 4-persona design also constrained our ability to evaluate the system across the full clinically relevant range of MADRS severity and across factorial combinations of demographic and stylistic features. Future research including a fully crossed design that varies severity (eg, mild, moderate, and severe) alongside gender and other demographic characteristics would provide a more informative evaluation of system fidelity.

Beyond gender, demographic variables including race, ethnicity, education, and socioeconomic status were not systematically controlled in the persona configurations. Because LLMs can produce demographically patterned linguistic variation, this limits our ability to characterize how the system performs across diverse simulated patient backgrounds and represents an important target for future, prompt-engineered evaluations.

Two features of the rater sample warrant acknowledgment as limitations. First, although all raters were experienced MADRS administrators, no independent baseline calibration was conducted prior to the study; we therefore cannot distinguish scoring discrepancies arising from the simulation from those reflecting pre-existing rater drift. Second, raters were recruited through the authors’ professional network, which introduces the possibility of demand characteristics favorably biasing subjective ratings of realism and coherence. Future evaluations should incorporate independent baseline calibration of raters from a representative sample of clinical raters outside the authors’ professional network.

Moreover, the current simulations are based only on audio, lacking any nonverbal cues such as facial expressions and body language. These cues are an essential part of clinical assessment, for instance in evaluating items such as “apparent sadness” on the MADRS, and their absence limits the system’s ecological validity. Relatedly, a formal analysis of system response latency would contribute to a more in-depth understanding of the perception of natural dialogue produced by the model, particularly in the context of evaluating symptoms of depression. An important next step is to integrate the voice pipeline into a multimodal simulation, for instance with animated 3D avatars, that can train raters to interpret both verbal and nonverbal communication, while also accounting for system factors such as response latency.

While the virtual patients in our simulations were rated as highly narratively cohesive, it is important to acknowledge that LLMs are inherently susceptible to generating factually inconsistent or clinically implausible content. Even though no such failures were reported by raters in this controlled study, this reflects postinterview impressions rather than independent transcript review, and robust guardrails and continuous validation will be essential for deploying such a system in a real-world training environment.

In addition, a more formal examination of the observed systematic bias at the persona-level, rater-level, and interactions thereof would help to identify the source of the observed overestimation. Relatedly, a more in-depth transcript analysis to interrogate any factual and clinical inconsistencies contributing to bias was not included in the present study and would be an important inclusion for future work. Although the current sample size precludes the stable estimation of variance components, expanding this sample will be an important avenue for future work—particularly to rule out instability arising from LLM nondeterminism.

The present study focused on symptoms of depression as measured specifically by the MADRS. In practice, this tool holds promise for supporting clinical assessment training across a broad range of psychiatric conditions and assessment scales, such as those for anxiety and bipolar disorder. Careful validation will be necessary to demonstrate the system’s generalizability to these scenarios.

Finally, a critical future direction will be to rigorously test the system’s pedagogical effectiveness in a real training scenario. While expert feedback suggests that the tool would be beneficial, a longitudinal study is required to empirically determine whether training with this simulation system can lead to demonstrable improvements in trainee scoring accuracy, confidence, and interviewing skills compared with traditional training methods.

### Conclusions

LLM-powered virtual patient simulations represent a promising proof-of-concept tool for training clinicians in standardized clinical assessments. This pilot study provides preliminary evidence that the system can produce clinically plausible practice scenarios that experts can score with reasonable fidelity and with promising, though preliminary, reliability estimates. Future studies should continue to address the empirical gap in how this training tool improves data quality in clinical trials above and beyond current training approaches. By addressing the need for scalable, standardized, and realistic training opportunities, this technology has the potential to enhance the quality of psychiatric assessments, improve data integrity in mental health clinical trials, and support scalable rater training for psychiatric assessment. Future work will focus on expanding the system’s capabilities and rigorously testing its impact on trainee performance over time to solidify its role in medical education.

## Abbreviations

CNS: central nervous system
ICC: intraclass correlation coefficient
ISCTM: International Society for CNS Clinical Trials and Methodology
LLM: large language model
MADRS: Montgomery-Åsberg Depression Rating Scale
SIGMA: Structured Interview Guide for the Montgomery-Åsberg Depression Rating Scale
STT: speech-to-text
TTS: text-to-speech
